# Differences in prevalence of prehypertension and hypertension in children and adolescents in the eastern, central and western regions of China from 1991-2011 and the associated risk factors

**DOI:** 10.1371/journal.pone.0210591

**Published:** 2019-01-10

**Authors:** Zhaoyang Fan, Zijun Liao, Xinnan Zong, Shuaiming Zhang

**Affiliations:** 1 Department of Early Child Development, Capital Institute of Pediatrics, Beijing, China; 2 Institute of Reproductive & Child Health/ Ministry of Health Key Laboratory of Reproductive Health, Peking University Health Science Center; Department of Epidemiology and Biostatistics, School of Public Health, Peking University Health Science Center, Beijing, China; 3 Department of Growth, Capital Institute of Pediatrics, Beijing, China; University of Utah School of Medicine, UNITED STATES

## Abstract

The present study aimed to estimate the differences in rates of prehypertension and hypertension in children and adolescents among three regions with different socioeconomic status in China, and explore the corresponding risk factors associated with prehypertension and hypertension to guide the prevention. Blood pressure measurements of 13 762 children and adolescents aged 6–17 years were obtained from a prospective national survey (the China Health and Nutrition Survey, 1991–2011). Prehypertension and hypertension were defined by age and gender, according to China’s standard criteria. Chi-square tests were used to compare the differences in the prevalence of prehypertension and hypertension among three regions. Trend chi-square tests were used to detect the trends in rates of prehypertension and hypertension over survey years. Logistic regression models were used to detect the potential risk factors of prehypertension and hypertension in children and adolescents. During the survey years, the overall prevalence of prehypertension and hypertension were 6.0% and 10.6%. The corresponding rates in the western region were lowest, but increased rapidly over the two decades (84.0% and 122.6% increases respectively, *P*<0.001). The overall hypertension rate remained high in the eastern region, despite the slower increase (24.2% increase). In the central region, although the prehypertension rate remained stable, the rate of hypertension had a 94.8% increase these years (*P*<0.0001). According to the results of logistic regression, age, body mass index (BMI) and waist/height ratio (WHtR) were associated with prehypertension and hypertension. Children and adolescents in the eastern region had the highest level of prehypertension and hypertension, while the rapid increase of blood pressure in the western and central regions were also supposed to concern. Improvement of the healthy lifestyle is urgent for prehypertension and hypertension prevention in children and adolescents.

## Introduction

Adult hypertension has been epidemic worldwide and considered as an important risk factor for chronic diseases, such as cardiovascular disease [1]. As a systematic analysis showed, 31.3% of adults in the world had hypertension in 2010 [2]. In China, 27.8% of adults were hypertensive in 2013–2014 [3]. It has been found that the origin of hypertension in adulthood extends back to childhood [4]. Levels of blood pressure have been observed to track over time, and children with elevated blood pressure are more likely to become hypertensive adults [5–6]. Therefore, hypertension among children has become a significant public health issue in the last decades [7–10].

In China, there are various researches concerned on blood pressure in children and adolescents [11–15]. However, a majority of these studies are with limited time and geographic coverage. Socioeconomic status is associated with hypertension [16–18], although the results were ambivalent. Given the marked diversity of economy throughout China, it is necessary to estimate differences of prehypertension and hypertension in areas with varied economic development level. What’s more, the population pattern of hypertension is not static, since it rises and falls over time. A prior survey showed that the prevalence of hypertension in Chinese children and adolescents increased in the recent two decades [19], whereas the trends in the three regions in China remains unclear and they are worthy of exploring, considering the increasingly rapid economic growth and the uneven urbanization in China in recent decades.

Additionally, although a few studies have been conducted to evaluate the risk factors of prehypertension and hypertension in children and adolescents of China [7, 11], few studies were trying to explore the risk factors in different regions, given the different geography, economy and life circumstances.

This study aimed to estimate the differences in the prevalence of prehypertension and hypertension in children and adolescents among the eastern, central and western regions with different socioeconomic status in China, and explore risk factors associated with prehypertension and hypertension by three regions to guide the prevention and control of prehypertension and hypertension. Our data were extracted from the China Health and Nutrition Surveys (CHNSs) conducted during 1991–2011.

## Methods

### Study population

Data from CHNSs, conducted in 1991, 1993, 1997, 2000, 2004, 2006, 2009 and 2011, were extracted for nine provinces (i.e. Heilongjiang, Liaoning, Shandong, Jiangsu, Henan, Hubei, Hunan, Guangxi and Guizhou). The design and method of this survey were described in detail at the CHNSs website [20, 21]. In brief, those surveys used a stratified multistage random-cluster sampling method to select the sample surveyed in each of the provinces.

Data collection were reviewed and approved by the University of North Carolina and the China Centre for Disease Control and Prevention. The current study was approved by the Ethics Committee of Capital Institute of Paediatrics. All the data were collected from public online sources. All data were fully anonymized and no identifiable information was collected.

### Measurements and definitions

The measurements were same with a prior study [7]. Blood pressure was measured by trained examiners using a mercury sphygmomanometer according to a standard protocol [22], and was measured three times on one visit. The three measurements were separated by at least a 1 to 2-min interval, during which the right arm was raised up for 5–6 s. Korotkoff phase 1 and Korotkoff phase 5 were used for defining systolic blood pressure (SBP) and diastolic blood pressure (DBP) respectively. The average of blood pressure values of three times was reported.

The criteria for pre-hypertension and hypertension were according to the age- and gender-specific blood pressure reference standard for Chinese children and adolescents [23]. Hypertension was defined as average SBP and/or DBP that is ≥95th percentile for gender and age, and pre-hypertension in children was defined as average SBP or DBP levels that are ≥90th percentile but <95th percentile.

Participants’ age was dichotomised into 6–12 year (children) and 13–17 year (adolescents) [24], and both genders (boy and girl) were recruited. Body mass index (BMI) was calculated as weight in kilograms divided by the square of height in meters. The criteria to define overweight and obesity was proposed by the IOTF using age- and gender-specific BMI reference standard [25]. Abdominal obesity was defined as WHtR (waist/height ratio, calculated as waist circumferences divided height) ≥0.5 [26]. The regions of surveyed provinces were divided into three parts (eastern, central and western regions), as defined by the National Bureau of Statistics of China according to sociodemographic status, in which the eastern region included Liaoning, Jiangsu and Shandong, the central region included Heilongjiang, Henan, Hubei, and Hunan, and the western region included Guizhou and Gansu [27]. Location was divided into urban and rural areas. The urbanization index was collected and divided into quartiles, which consists of 12 components [28]. Information of 3-day average energy, carbohydrate, fat and protein were collected and divided into quartiles.

### Statistical analysis

Mean (SD) or median [interquartile ranges (IQR)] was used to present continuous variable and frequencies (%) to present categorical variable. Significant differences among the variables are determined by analysis of variance (ANOVA) for normally distributed variables, Kruskal-Wallis tests for skewed distributed variables, and chi-square tests for categorical data accordingly. Additionally, the differences across the three regions were following tested by Bonferroni adjustments for multiple comparisons. For each of the 3 comparisons, a significance level of *P* = 0.0167 (0.05/3) provided an overall type I error rate of 0.05. Trend chi-square test was used to detect the trends in rates of prehypertension and hypertension over survey years. Logistic regression models were used to detect the associations between the prevalence of prehypertension and hypertension and potential risk factors, including year (1991–1997, 2000–2006 and 2009–2011), location (urban and rural areas), urbanization index [Q1 (the lowest quartile), Q2, Q3 and Q4 (the highest quartile)], age (6–12, 13–17 years), sex (male, female), BMI (thin and normal, overweight and obesity), abdominal obesity (no, yes), tobacco consumption (no, yes), alcohol consumption (no, yes), 3-day average energy (Q1, Q2, Q3 and Q4), 3-day average carbohydrate (Q1, Q2, Q3 and Q4), 3-day average fat (Q1, Q2, Q3 and Q4), 3-day average protein (Q1, Q2, Q3 and Q4).

## Results

### Characteristics of participants

From 1991 to 2011, 16 181 children and adolescents were recruited for surveys, among whom 13 762 (85.1%) participants were included with blood pressure values were available. The population characteristics were comparable between children and adolescents included in our study and those excluded (*P*>0.05), except those excluded from this analysis were older (*P*<0.001). Among the 13 762 children and adolescents, 4030 (29.3%), 5476 (39.8%) and 4256 (30.9%) were respectively from the eastern, central and western regions. 3899 (28.3%) were in urban areas, while 9863 (71.7%) were in rural areas. The mean age was 11.7 (3.1) years, with 8052 (58.5%) and 5710 (41.5%) were respectively children and adolescents. 7205 (52.4%) were male, and 6557 (47.7%) were female. Those overweight accounted for 6.7% (902), and 251 (1.9%) were obesity. The characteristics of participants according to the three regions are shown in **Table 1**. The mean age and proportion of male were similar among the three regions (*P*>0.05), while the distributions of obesity status, urban residence, urbanisation index and the life risk factors differed significantly by region (*P*<0.0001). The eastern region, as the most developed area in China, was with the highest urbanisation index, the largest proportions of urban residents as well as children and adolescents who were overweight or obesity. The children and adolescents in the western region were with higher tobacco and alcohol consumption, and lower energy, carbohydrate, fat and protein intake, compared with those in the eastern and central regions.

**Table 1:** Characteristics of participants stratified by region.

Characteristics	Region			*P*
	Eastern	Central	Western	
**Year**				<0.0001
1991	777 (19.3)	841 (15.4)	746 (17.5)	
1993	745 (18.5)	867 (15.8)	654 (15.4)	
1997	451 (11.2)	1145 (20.9)	669 (15.7)	
2000	656 (16.3)	1001 (18.3)	544 (12.8)	
2004	385 (9.6)	550 (10.0)	364 (8.6)	
2006	268 (6.7)	480 (8.8)	373 (8.8)	
2009	246 (6.1)	278 (5.1)	366 (8.6)	
2011	502 (12.5)	314 (5.7)	540 (12.7)	
**Location**				<0.0001
Urban	1063 (26.4)	1538 (28.1)	1298 (30.5)	
Rural	2967 (73.6)	3938 (71.9)	2958 (69.5)	
**Urbanisation index**				<0.0001
Q1 (the lowest quartile)	795 (19.7)	1503 (27.5)	1142 (26.8)	
Q2	861 (21.4)	1538 (28.1)	1036 (24.3)	
Q3	1161 (28.8)	1293 (23.6)	994 (23.4)	
Q4 (the highest quartile)	1213 (30.1)	1142 (20.9)	1084 (25.5)	
**Age, y**				0.037
Children (6~12)	2295 (57.0)	3262 (59.6)	2495 (58.6)	
Adolescents (13~17)	1735 (43.1)	2214 (40.4)	1761 (41.4)	
**Sex**				0.436
Male	2093 (51.9)	2849 (52.0)	2263 (53.2)	
Female	1937 (48.1)	2849 (48.0)	1993 (46.8)	
**Blood Pressure**				<0.0001
Normotension	3199 (79.4)	4503 (82.2)	3770 (88.6)	
Prehypertension	266 (6.6)	365 (6.7)	200 (4.7)	
Hypertension	565 (14.0)	608 (11.1)	286 (6.7)	
**Obesity status**				<0.0001
Thin and Normal	3334 (85.3)	4938 (92.3)	4022 (96.1)	
Overweight	449 (11.5)	324 (6.1)	129 (3.1)	
Obesity	126 (3.2)	89 (1.7)	36 (0.9)	
**Abdominal obesity**				<0.0001
No	2477 (88.1)	3715 (92.5)	3042 (94.4)	
Yes	334 (11.9)	303 (7.5)	179 (5.6)	
**Tobacco consumption**				<0.0001
No	4004 (99.4)	5418 (98.9)	4175 (98.1)	
Yes	26 (0.7)	58 (1.1)	81 (1.9)	
**Alcohol consumption**				<0.001
No	3924 (97.4)	5316 (97.1)	4080 (95.9)	
Yes	106 (2.6)	160 (2.9)	176 (4.1)	
**3-day average energy, kcal**				<0.0001
Q1 (the lowest quartile)	989 (24.5)	1263 (23.1)	1188 (27.9)	
Q2	958 (23.8)	1363 (24.9)	1120 (26.3)	
Q3	1061 (26.3)	1400 (25.6)	980 (23.0)	
Q4 (the highest quartile)	1022 (25.4)	1450 (26.5)	968 (22.7)	
**3-day average carbohydrate, g**				<0.0001
Q1 (the lowest quartile)	1185 (29.4)	1108 (20.2)	1147 (27.0)	
Q2	961 (23.9)	1426 (26.0)	1054 (24.8)	
Q3	925 (23.0)	1485 (27.1)	1031 (24.2)	
Q4 (the highest quartile)	959 (23.8)	1457 (26.6)	1024 (24.1)	
**3-day average fat, g**				<0.0001
Q1 (the lowest quartile)	870 (21.6)	1485 (27.1)	1085 (25.5)	
Q2	1000 (24.8)	1330 (24.3)	1111 (26.1)	
Q3	1035 (25.7)	1313 (24.0)	1093 (25.7)	
Q4 (the highest quartile)	1125 (27.9)	1348 (24.6)	967 (22.7)	
**3-day average protein, g**				<0.0001
Q1 (the lowest quartile)	776 (19.3)	1363 (24.9)	1301 (30.6)	
Q2	881 (21.9)	1437 (26.2)	1123 (26.4)	
Q3	1093 (27.1)	1369 (25.0)	979 (23.0)	
Q4 (the highest quartile)	1280 (31.8)	1307 (23.9)	853 (20.0)	
**Urbanisation index, median (IQR)**	55.3 (41.3–73.5)	47.9 (37.8–65.1)	50.4 (38.0–68.8)	<0.0001
**Age, y, mean (SD)**	11.8 (3.1)	11.7 (3.0)	11.7 (3.1)	0.080
**BMI, kg/m**^**2**^**, median (IQR)**	18.0 (16.0–20.3)	17.1 (15.4–19.2)	16.4 (15.0–18.5)	<0.0001
**SBP, mmHg, median (IQR)**	100.0 (90.0–110.0)	99.3 (90.0–108.0)	94.3 (88.7–102.7)	<0.0001
**DBP, mmHg, median (IQR)**	66.0 (60.0–70.7)	64.0 (60.0–70.0)	60.7 (58.0–69.3)	<0.0001

Values are shown as n (%), unless indicated otherwise. IQR, interquartile range; SD, standard deviation; Q, quartile.

### Prevalence of prehypertension and hypertension

Children and adolescents were classified into three groups, which were normotensive, prehypertension and hypertension, according to the level of blood pressure. The overall prevalence of prehypertension and hypertension in children and adolescents was 6.0% and 10.6%, respectively. Across the study period, the overall prevalence of prehypertension and hypertension in children and adolescents was significantly different in the regions (*P*<0.0001). For the prehypertension of all survey years, the rates in the eastern, central and western regions were 6.6%, 6.7% and 4.7%. Under multiple comparisons, the overall prehypertension rates in the eastern and central regions were similar (*P*>0.0167), but significantly higher than that in the western region (*P*<0.0167). The hypertension rate was highest in the eastern region (14.0%), following by that in the central region (11.1%), and that in the western region was the lowest (6.7%). In 1991, the rates of prehypertension in the eastern, central and western regions were 4.3%, 6.1% and 2.8%, respectively (*P* = 0.007); the rates of hypertension in these corresponding regions were 11.1%, 7.9% and 4.2% (*P*<0.0001). In 2011, the rates of prehypertension in the corresponding regions were 10.8%, 4.8% and 5.2% (*P*<0.001); the hypertension rates were respectively 13.8%, 15.3% and 9.3% (*P* = 0.017).

**Fig 1:**
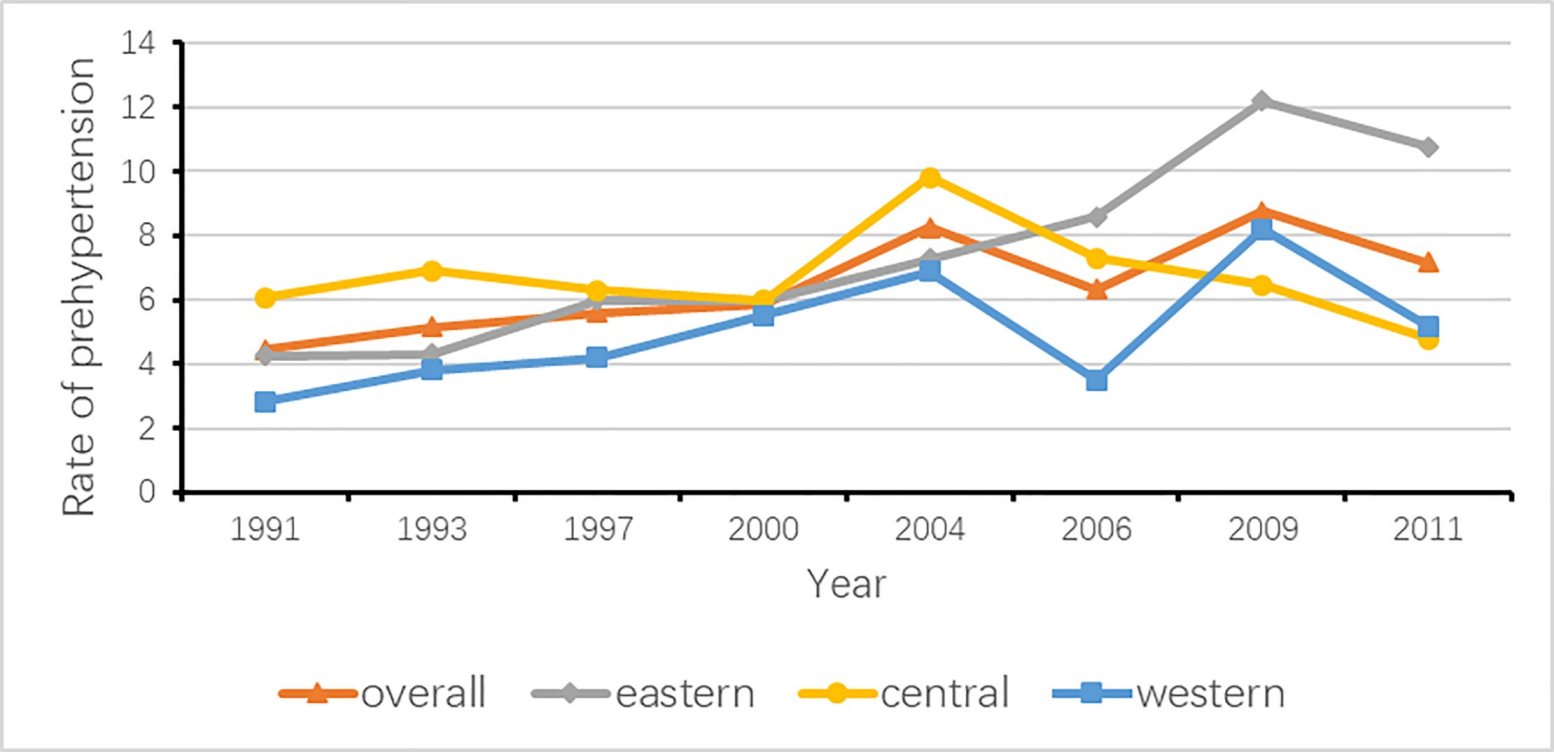
Trends of prehypertension by survey years.

**Fig 2:**
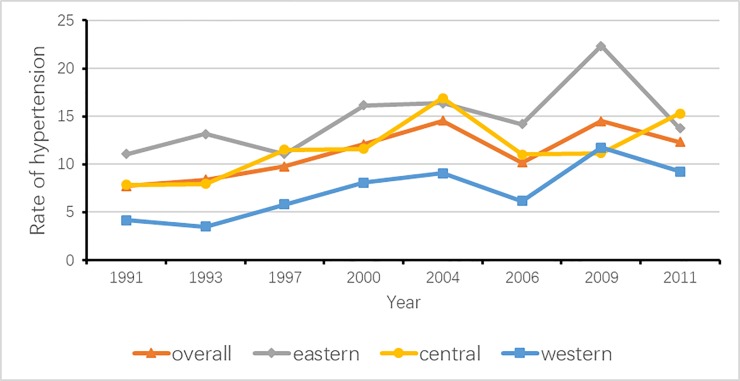
Trends of hypertension by survey years.

In addition, as **Fig 1** and **Fig 2** show, the yearly total prevalence of prehypertension and that of hypertension, as well as the corresponding prevalence according to the three regions, were compared to explore the variation tendency. From 1991 to 2011, the trajectories in the rates of prehypertension and hypertension were similar, except the trend of prehypertension in the central region. The total prevalence of prehypertension significantly increased from 4.4% to 7.2% (*P*<0.0001), and the prevalence of hypertension increased from 7.7% to 12.3% (*P*<0.0001). For children and adolescents in the eastern region, the prevalence of prehypertension increased from 4.3% in 1991 to 10.8% in 2011 (*P*<0.0001), and prevalence of hypertension increased from 11.1% in 1991 to 22.4% in 2009, and then decreased to 13.8% (*P* = 0.001). For those in the central region, the incidence of prehypertension remained stable from 6.1% in 1991 to 4.8% in 2011 (*P* = 0.589), while the rate of hypertension increased from 7.9% in 1991 to 15.3% in 2011 (*P*<0.0001). The prehypertension prevalence for those in the western region significantly rose from 2.8% in 1991 to 5.2% in 2011 (*P* = 0.001); in the meantime, the corresponding hypertension rate significantly increased from 4.2% in 1991 to 9.3% in 2011 (*P*<0.0001).

### Logistic regression analyses

The potential factors of prehypertension and hypertension versus normotension in children and adolescents were explored. **Table 2** showed the general results of crude and adjusted ORs for these potential factors. A few associations were still significant after adjustments. After adjustments, we found that adolescents were at a higher risk of prehypertension and hypertension than children (adjusted OR: 2.483; 95%CI: 2.213, 2.786; *P*<0.0001). Overweight children and adolescents had nearly 2 fold increased risk of developing prehypertension and hypertension (adjusted OR: 1.954; 95%CI: 1.619, 2.357; *P*<0.0001) and obese children and adolescents had more than 2 fold increased risk of developing prehypertension and hypertension compared with those with healthy or low weight (adjusted OR: 2.427; 95%CI: 1.757, 3.354; *P*<0.0001); a similar pattern existed for abdominal obesity (adjusted OR: 1.706; 95%CI: 1.421, 2.049; *P*<0.0001).

**Table 2:** Factors associated with prehypertension and hypertension.

Variables	Crude OR (95%CI)	*P*	Adjusted OR (95%CI)	*P*
**Year**				
1991~1997	Ref			
2000~2006	1.278 (1.165, 1.403)	<0.0001	1.271 (1.115, 1.447)	<0.001
2009~2011	1.415 (1.263, 1.585)	<0.0001	1.452 (1.234, 1.708)	<0.0001
**Location**				
Urban	Ref		Ref	
Rural	0.816 (0.740, 0.899)	<0.0001	0.938 (0.827, 1.065)	0.324
**Urbanization index**				
Q1 (the lowest quartile)	Ref		Ref	
Q2	1.047 (0.944, 1.160)	0.385	1.041 (0.878, 1.233)	0.645
Q3	0.919 (0.828, 1.021)	0.116	0.895 (0.752, 1.065)	0.212
Q4 (the highest quartile)	1.422 (1.288, 1.569)	<0.0001	1.008 (0.837, 1.215)	0.933
**Age, y**				
Child (6~12)	Ref		Ref	
Adolescent (13~17)	2.518 (2.297, 2.760)	<0.0001	2.483 (2.213, 2.786)	<0.0001
**Sex**				
Male	Ref		Ref	
Female	1.006 (0.920, 1.101)	0.894	1.083 (0.970, 1.207)	0.155
**BMI, kg/m**^**2**^				
Thin and Normal	Ref		Ref	
Overweight	2.135 (1.834, 2.485)	<0.0001	1.954 (1.619, 2.357)	<0.0001
Obesity	2.438 (1.864, 3.189)	<0.0001	2.427 (1.757, 3.354)	<0.0001
**Abdominal obesity**				
No	Ref		Ref	
Yes	2.320 (1.983, 1.983)	<0.0001	1.706 (1.421, 2.049)	<0.0001
**Tobacco consumption**				
No	Ref		Ref	
Yes	1.780 (1.254, 2.528)	0.001	1.157 (0.740, 1.811)	0.522
**Alcohol consumption**				
No	Ref		Ref	
Yes	2.008 (1.624, 2.484)	2.484	1.135 (0.854, 1.510)	0.383
**3-day average energy, kcal**				
Q1 (the lowest quartile)	Ref		Ref	
Q2	0.850 (0.764, 0.946)	0.003	1.088 (0.888, 1.332)	0.416
Q3	1.067 (0.963, 1.182)	0.216	1.158 (0.871, 1.541)	0.313
Q4 (the highest quartile)	1.316 (1.192, 1.454)	<0.0001	1.110 (0.763, 1.613)	0.586
**3-day average carbohydrate, g**				
Q1 (the lowest quartile)	Ref		Ref	
Q2	0.891 (0.801, 0.990)	0.032	0.927 (0.778, 1.103)	0.392
Q3	0.925 (0.833, 1.027)	0.145	0.895 (0.711, 1.127)	0.345
Q4 (the highest quartile)	1.227 (1.110, 1.357)	<0.0001	1.072 (0.792, 1.451)	0.652
**3-day average fat, g**				
Q1 (the lowest quartile)	Ref		Ref	
Q2	0.976 (0.880, 1.083)	0.651	1.084 (0.920, 1.279)	0.335
Q3	1.035 (0.934, 1.148)	0.509	1.108 (0.927, 1.326)	0.261
Q4 (the highest quartile)	1.296 (1.173, 1.432)	1.432	1.058 (0.845, 1.325)	0.621
**3-day average protein, g**				
Q1 (the lowest quartile)	Ref		Ref	
Q2	0.840 (0.755, 0.935)	0.001	0.936 (0.788, 1.112)	0.454
Q3	0.990 (0.892, 1.098)	0.850	0.948 (0.778, 1.155)	0.594
Q4 (the highest quartile)	1.472 (1.335, 1.624)	<0.0001	1.067 (0.852, 1.335)	0.575

OR, odds ratio; CI, confidence interval; Ref, reference; Q, quartile.

In crude analyses, living in rural areas decreased the corresponding risk (crude OR: 0.816; 95%CI: 0.740, 0.899; *P* < .0001), while living in the area with high urbanization index increased the risk (crude OR: 1.422; 95%CI: 1.288, 1.569; *P*<0.0001). Superfluous energy, carbohydrate and protein intake (Q4) were risk factors (*P*<0.0001), while the corresponding medium intake (Q2) were protective factors (*P*<0.05). However, these associations mentioned above in crude analyses became non-significant after adjustments (*P*>0.05).

In the three regions, age and obesity were still associated with increased prehypertension and hypertension. (*P*<0.01, **Table 3**). Besides, after adjustments, superfluous carbohydrate intake was positively associated with prehypertension and hypertension in children and adolescents residing in the eastern region (OR: 1.733; 95%CI: 1.019, 2.948; *P*<0.0001), and alcohol consumption increased the corresponding risk (OR: 1.693; 95%CI: 1.089, 2.630; *P*<0.0001) in those living in the western region.

**Table 3:** Factors associated with prehypertension and hypertension in three regions.

Variables	Eastern		Central		Western	
	Crude OR (95%CI)	Adjusted OR (95%CI)	Crude OR (95%CI)	Adjusted OR (95%CI)	Crude OR (95%CI)	Adjusted OR (95%CI)
**Year**						
1991–1997	Ref					
2000–2006	1.202 (1.024, 1.411)	1.508 (1.17, 1.943)	1.301 (1.130, 1.499)	0.994 (0.825, 1.197)	1.261 (1.033, 1.033)	1.596 (1.197, 2.129)
2009–2011	1.644 (1.370, 1.973)	1.892 (1.393, 2.568)	1.090 (0.876, 1.356)	0.954 (0.726, 1.254)	1.800 (1.462, 2.216)	2.277 (1.637, 3.165)
**Location**						
Urban	Ref					
Rural	0.690 (0.584, 0.814)	0.822 (0.64, 1.055)	0.861 (0.740, 1.001)	1.062 (0.868, 1.298)	0.854 (0.698, 1.043)	0.903 (0.707, 1.152)
**Urbanization index**						
Q1 (the lowest quartile)	Ref					
Q2	0.986 (0.818, 1.189)	0.960 (0.668, 1.38)	1.101 (0.945, 1.282)	1.355 (1.065, 1.723)	1.060 (0.853, 1.318)	0.861 (0.612, 1.211)
Q3	0.891 (0.751, 1.057)	0.791 (0.562, 1.113)	0.882 (0.746, 1.042)	1.073 (0.832, 1.384)	0.930 (0.741, 1.167)	0.792 (0.553, 1.135)
Q4 (the highest quartile)	1.575 (1.342, 1.848)	0.965 (0.654, 1.426)	1.316 (1.118, 1.549)	1.235 (0.934, 1.633)	1.326 (1.078, 1.631)	0.775 (0.537, 1.117)
**Age, y**						
Child (6~12)	Ref					
Adolescent (13~17)	2.186 (1.872, 2.552)	2.047 (1.672, 2.506)	2.422 (2.103, 2.789)	2.346 (1.969, 2.796)	3.494 (2.856, 4.276)	3.456 (2.696, 4.431)
**Sex**						
Male	Ref					
Female	0.997 (0.856, 1.162)	1.150 (0.947, 1.395)	1.101 (0.958, 1.265)	1.130 (0.955, 1.336)	0.840 (0.694, 1.016)	0.893 (0.711, 1.12)
**BMI**						
Thin and Normal	Ref					
Overweight	1.895 (1.526, 2.352)	1.666 (1.262, 2.199)	1.767 (1.368, 2.283)	1.609 (1.176, 2.200)	2.553 (1.685, 3.868)	2.803 (1.708, 4.602)
Obesity	1.830 (1.248, 2.685)	1.578 (0.972, 2.562)	2.522 (1.622, 3.923)	2.372 (1.404, 4.007)	3.026 (1.450, 6.314)	4.580 (1.966, 10.668)
**Abdominal obesity**						
No	Ref					
Yes	2.987 (2.352, 3.793)	2.430 (1.834, 3.219)	1.850 (1.425, 2.401)	1.539 (1.140, 2.078)	1.447 (0.968, 2.161)	0.887 (0.553, 1.424)
**Tobacco consumption**						
No	Ref					
Yes	1.156 (0.463, 0.463)	0.854 (0.272, 2.681)	2.103 (1.2, 3.684)	1.409 (0.692, 2.868)	2.434 (1.443, 4.105)	1.096 (0.556, 2.16)
**Alcohol consumption**						
No	Ref					
Yes	1.540 (1.002, 2.366)	1.003 (0.528, 1.902)	2.098 (1.488, 2.959)	0.881 (0.545, 1.425)	2.835 (1.992, 4.034)	1.693 (1.089, 2.630)
**3-day average energy, kcal**						
Q1 (the lowest quartile)	Ref					
Q2	0.868 (0.722, 1.043)	0.992 (0.689, 1.427)	0.946 (0.805, 1.112)	1.260 (0.919, 1.728)	0.708 (0.563, 0.892)	1.039 (0.689, 1.567)
Q3	1.010 (0.849, 1.200)	1.000 (0.602, 1.659)	1.090 (0.932, 1.275)	1.368 (0.878, 2.132)	1.028 (0.822, 1.285)	1.162 (0.644, 2.095)
Q4 (the highest quartile)	1.383 (1.169, 1.637)	0.998 (0.513, 1.945)	1.116 (0.956, 1.302)	1.184 (0.661, 2.120)	1.558 (1.265, 1.919)	1.383(0.647, 2.955)
**3-day average carbohydrate, g**						
Q1	Ref					
Q2	0.902 (0.752, 1.082)	1.103 (0.809, 1.503)	0.940 (0.802, 1.103)	0.960 (0.732, 1.259)	0.786 (0.625, 0.99)	0.779 (0.540, 1.126)
Q3	0.911 (0.757, 1.095)	1.244 (0.828, 1.868)	0.945 (0.808, 1.106)	0.872 (0.607, 1.253)	0.905 (0.905, 1.133)	0.691 (0.425, 1.125)
Q4	1.277 (1.073, 1.518)	1.733 (1.019, 2.948)	1.156 (0.991, 1.348)	1.034 (0.644, 1.661)	1.291 (1.045, 1.594)	0.765 (0.408, 1.433)
**3-day average fat, g**						
Q1 (the lowest quartile)	Ref					
Q2	1.006 (0.844, 1.201)	1.285 (0.934, 1.767)	1.011 (0.861, 1.189)	1.007 (0.789, 1.286)	0.908 (0.729, 1.13)	1.036 (0.74, 1.451)
Q3	0.989 (0.83, 1.178)	1.339 (0.952, 1.885)	1.068 (0.910, 1.254)	1.020 (0.779, 1.335)	1.064 (0.86, 1.318)	1.099 (0.765, 1.579)
Q4 (the highest quartile)	1.357 (1.151, 1.600)	1.447 (0.960, 2.181)	1.101 (0.940, 1.29)	0.899 (0.634, 1.274)	1.457 (1.181, 1.799)	0.936 (0.596, 1.471)
**3-day average protein, g**						
Q1 (the lowest quartile)	Ref					
Q2	0.835 (0.690, 1.011)	0.793 (0.568, 1.107)	0.910 (0.776, 1.068)	0.851 (0.654, 1.107)	0.001 (0.628, 0.984)	1.039 (0.735, 1.468)
Q3	0.908 (0.763, 1.081)	0.760 (0.525, 1.101)	1.005 (0.857, 1.179)	0.856 (0.628, 1.167)	1.003 (0.801, 1.255)	0.971 (0.647, 1.457)
Q4 (the highest quartile)	1.461 (1.246, 1.712)	0.790 (0.52, 1.201)	1.219 (1.042, 1.428)	0.919 (0.645, 1.31)	1.674 (1.352, 2.073)	1.267 (0.794, 2.023)

OR, odds ratio; CI, confidence interval; ref, reference; Q, quartile.

## Discussion

We examined the differences in prehypertension and hypertension rates in children and adolescents according to the eastern, central and western regions in China. For the whole study period, the rates of prehypertension and hypertension were 6.0% and 10.6%, respectively; the prevalence of prehypertension in the eastern and central regions was significantly higher than that in the western region; and the hypertension rate was highest in the eastern region, followed by central region, and that in the western region was lowest. From 1991 to 2011, there were increasing trajectories in the rates of prehypertension and hypertension in the three regions, except the trend of prehypertension rate in the central region.

Children and adolescents in the eastern region had the highest prevalence of pre-hypertension and hypertension over the two decades. According to the Chinese government, the eastern region has been the most developed and has the highest socioeconomic status, maybe leading to the children and adolescents’ intake of more modern convenient diets that are high in sodium and saturated fats and low in vegetables [29], which is an essential factor of blood pressure elevation [30]. In the present study, the rate of prehypertension in the eastern region was 4.3% in 1991 and 10.8% in 2011 respectively, with a 153% increase, while the rate of hypertension in the eastern region increased 24% from 11.1% in 1991 to 13.8% in 2011. The relatively less increase of hypertension rate might result from rising attention toward blood pressure and the implement of lifestyle interventions [31, 32]. What’s more, in the eastern region, although the prehypertension rate was not higher than the hypertension rate, the rate of prehypertension increased rapidly in recent decades. Prehypertension has accounted in no small degree as a precursor of hypertension and has been associated with excess morbidity and mortality from cardiovascular diseases [33]. This rapid increase suggests that much more attention should be given to prehypertension control, such as the lifestyle and frequent blood pressure monitoring necessary to treat prehypertension [34]. In the central region, the prevalence of prehypertension remained stable; on the contrary, its hypertension rate increased from 7.9% to 15.3%, with nearly twice in two decades. Besides, in the western region, the rates of prehypertension and hypertension increased rapidly as well, with 84.0% and 122.6% increase respectively, despite the remaining lowest prevalence of prehypertension and hypertension among the three regions. These variations were in accordance with the development of the economy [35], and the trends in overall blood pressure indicated that controlling blood pressure in relatively less developed areas became a public concern as well. Consistent with the increasing upward trends of prevalence of prehypertension and hypertension reported by other studies [7, 8, 36], the nearly twice rates of prehypertension in the eastern and western regions, as well as hypertension in the central and western regions in our study, indicated the urgency of the high blood pressure prevention in children and adolescents after twenty years urbanization development in China.

We observed overweight and obese children and adolescents respectively had higher possibilities in developing prehypertension and hypertension, consistent with other studies that blood pressure or hypertension were positively correlated with BMI and obesity [11, 14]. The prevalence of overweight and obesity in children and adolescents increased across the latest decades [36, 37], with the enrichment of material consumption. The levels of blood pressure increased in parallel with the increasing BMI among Chinese boys and girls, possibly since some adipocyte-derived factors are linked to blood pressure control, and aberrant production and release of those factors may contribute to the high prevalence of hypertension in the obese population [36, 38, 39]. In this study, we also found the higher risk for adolescents to have prehypertension and hypertension than children, inconsistent with the previous report [7]. The possible reasons for our finding may be due to the complex physiological and hormonal changes during puberty, and sexual maturation during adolescence [40]. It is worth mentioning that the urban location was not significant after adjustments, which might be because the lifestyle, such as the consumption of energy-rich foods, expenditure of energy and social support, was the more important reason. Additionally, alcohol consumption was found to be associated with prehypertension and hypertension in children and adolescents in the western regions, in which the rate of alcohol consumption was higher than that in the eastern and central areas, indicating the need to intervene in the unhealthy lifestyle in these target population, especially in adolescents.

There are some strengths in our study. First, we stratified and analysed the data from three regions with different sociodemographic statuses, which enabled us to estimate the blood pressure situation in regions with different socioeconomic developmental levels. Second, we used China’s standard criteria to identify children and adolescents with prehypertension and hypertension, which made our results comparable with other studies in China. Several limitations also existed in this study. First was that three measurements performed in one visit were used to calculate the mean SBP and DBP and judge the presence of prehypertension and hypertension, which might make the prevalence overrated. Second, causal links could not be evaluated, and some confounding factors could not be avoided since data came from the cross-sectional survey. Furthermore, we only looked at the factors with available data, so other important factors such as the family history of hypertension need to be investigated in future studies.

## Conclusions

Children and adolescents in the eastern region had the highest level of prehypertension and hypertension, while the rapid increase of blood pressure in the western and central regions were also supposed to concern. Improvement of the healthy lifestyle is urgent for prehypertension and hypertension prevention in children and adolescents.
